# Quintuply-fortified salt for the improvement of micronutrient status among women of reproductive age and preschool-aged children in Punjab, India: protocol for a randomized, controlled, community-based trial

**DOI:** 10.1186/s40795-022-00583-y

**Published:** 2022-09-06

**Authors:** Christine M. McDonald, Kenneth H. Brown, Yvonne E. Goh, Mari S. Manger, Charles D. Arnold, Nancy F. Krebs, Jamie Westcott, Julie M. Long, Rosalind S. Gibson, Manu Jamwal, Bidhi L. Singh, Neha Dahiya, Deepmala Budhija, Reena Das, Mona Duggal

**Affiliations:** 1 Departments of Pediatrics, and Epidemiology and Biostatistics, University of California, San Francisco, CA USA; 2 Department of Nutrition, and Institute for Global Nutrition, University of California, Davis, CA USA; 3 International Zinc Nutrition Consultative Group, Oakland, CA USA; 4 University of Colorado School of Medicine, Aurora, CO USA; 5 Department of Human Nutrition, University of Otago, Dunedin, New Zealand; 6 Postgraduate Institute of Medical Education and Research, Chandigarh, India; 7 Indian Council of Medical Research, Delhi, India

**Keywords:** Fortification, Micronutrients, Salt, Undernutrition

## Abstract

**Background:**

Multiple micronutrient (MN) deficiencies remain highly prevalent among women of reproductive age (WRA) and preschool-aged children (PSC) in many areas within India. Salt is an attractive vehicle for MN fortification in this context, as it is universally consumed in fairly consistent amounts and coverage of iodized salt (IS) is 94%. The overall objective of this trial is to evaluate the nutritional impact of quintuply-fortified salt with iron in the form of encapsulated ferrous fumarate, zinc, vitamin B12, folic acid, and iodine (eFF-Q5S) vs. quintuply-fortified salt with iron in the form of ferric pyrophosphate plus EDTA, zinc, vitamin B12, folic acid, and iodine (FePP-Q5S) vs. IS for the improvement of MN status among non-pregnant WRA and PSC.

**Methods:**

The study is a community-based, randomized, controlled trial that will be conducted in Punjab, India. 780 non-pregnant WRA 18–49 years old and 468 PSC 12–59 months old will be enrolled and assigned to one of three intervention groups. Salt will be provided to participants monthly for 12 months. Primary outcomes include changes in mean concentration of biomarkers of iron, zinc, vitamin B12, folate and iodine. Secondary outcomes include changes in the composition of the gut microbiome, and discretionary salt intake of PSC.

**Discussion:**

If proven efficacious, multiply-fortified salt (MFS) has the potential to drastically reduce the burden of MN deficiencies in India, and around the world. Although effectiveness research will be needed to examine the impact of MFS under programmatic conditions, salt fortification will piggy-back on existing platforms to produce IS and doubly-fortified salt (DFS), making it possible to scale-up the intervention quickly.

**Trial registration:**

Clinicaltrials.gov: NCT05166980; date of registration: December 22, 2021. Clinical Trials Registry-India: CTRI/2022/040332 and CTRI/2022/02/040333; date of registration: February 15, 2022.

## Background

The burden of micronutrient (MN) deficiencies among women of reproductive age (WRA) and preschool-aged children (PSC) remains unacceptably high in India [1, 2]. Biologically, WRA and PSC are particularly vulnerable to MN deficiencies given the need to replace women’s losses of some nutrients incurred through menstruation, pregnancy and lactation, and to provide adequate nutrients for children's rapid growth and development in utero and during the early years of life. Socioeconomic or cultural factors may also limit the quantity and/or quality of a woman or young child’s diet, further increasing the risk of deficiency. Results from India’s 2019–2021 National Family Health Survey (NFHS) indicate that 57% of non-pregnant WRA are anemic (hemoglobin < 12 g/dL) [2], and a recent survey in Haryana revealed that 75% of non-pregnant WRA had iron deficiency (serum ferritin < 15 ng/mL), 80% had folate insufficiency (red blood cell (RBC) folate < 748 nmol/L) and a consequently elevated risk of neural tube defects (NTDs) in their newborns, and 82% had vitamin B12 insufficiency (serum vitamin B12 < 301 pg/mL), placing them at risk for both hematological and neurological complications [3, 4]. The 2016–2018 Comprehensive National Nutrition Survey (CNNS) also provides evidence that multiple MN deficiencies are common among young children in India: 41% of PSC are anemic (hemoglobin < 11 g/dL), and the prevalence estimates of iron (inflammation-adjusted ferritin < 12 μg/dL), folate (RBC folate < 151 ng/mL), zinc (serum zinc < 65 μg/dL), and vitamin B12 deficiency (serum vitamin B12 < 203 pg/mL) are 32%, 23%, 19% and 14%, respectively [1].

Iron, zinc, vitamin B12, and folate are critically important for several biological processes related to a healthy pregnancy and the optimal growth and development of the fetus and offspring. Iron is required for energy production, oxygen transport, cellular proliferation, and pathogen destruction [5]. Zinc is an essential mineral for immune health, reproductive function, growth and development [6]. Folate and vitamin B12 also play important roles in DNA synthesis, fetal growth and neurological development, in addition to many other functions [7]. Thus, deficiencies in these MNs are associated with several adverse health outcomes, including preterm and small-for-gestational births, NTDs, as well as child diarrhea and stunting. These health problems are widespread in India where more than 3.5 million infants are born preterm each year, 7% of children under 5 are reported to suffer from diarrhea in the previous 2 weeks, and 36% of children under 5 are stunted [2, 8]. A recent analysis estimated that the prevalence of NTDs in South Asia is 32 per 10,000 live births [9]. Clearly there is an urgent need to improve the MN status of WRA and PSC in India.

MN fortification of a staple food or condiment can be one of many effective strategies for improving the MN status of a population, as the approach is cost-effective, utilizes existing delivery systems, can deliver multiple MNs simultaneously, and does not require behavior change by the population. Salt is an attractive vehicle for multiple MN fortification in India, as it is universally consumed in fairly consistent amounts, and 94% of households already use adequately iodized salt (IS) [2]. Therefore, salt fortification will reach vulnerable populations who do not consume fortified wheat flour. Furthermore, India has recently scaled up the distribution of doubly-fortified salt (DFS; i.e. salt fortified with iron and iodine) through the Public Distribution System (PDS), Pradhan Mantri Poshan Shakti Nirman (PM-POSHAN) program, and Integrated Child Development Services (ICDS) in several states.

Extensive research conducted in diverse settings has shown that DFS significantly improves iron status among nutritionally vulnerable groups [10–14]. Until recently, salt fortification has been limited to iodine and iron. However, with new technology, it is now possible to fortify salt with multiple MNs, including zinc, vitamin B12, and folic acid, in addition to iron and iodine [15]. Preliminary stability, sensory and acceptability testing of the new MFS formulations have yielded promising results, but data on the efficacy of MFS in improving MN status and related health outcomes are very limited. Furthermore, preliminary evidence from an effectiveness study of DFS in Uttar Pradesh indicates some consumers may reject salt containing iron in the form of encapsulated ferrous fumarate (eFF) because of undesirable effects on food color [16]. Ferric pyrophosphate (FePP) in combination with ethylenediaminetetraacetic acid (EDTA) as an enhancer of absorption has been proposed as a superior iron fortificant as it is white in color and more closely resembles standard IS [11].

The overall objective of the present trial is to compare quintuply-fortified salt with iron in the form of encapsulated ferrous fumarate, zinc, vitamin B12, folic acid, and iodine (eFF-Q5S) vs. quintuply-fortified salt with iron in the form of ferric pyrophosphate plus EDTA, zinc, vitamin B12, folic acid, and iodine (FePP-Q5S) vs. IS for the improvement of MN status among non-pregnant WRA and PSC in Punjab, India. The change in the mean concentration of biomarkers of the following MNs, and the corresponding change in the prevalence of each MN deficiency, will be considered primary outcomes: iron: inflammation-adjusted serum ferritin and soluble transferrin receptor; anemia: hemoglobin; zinc: serum zinc; vitamin B12: serum vitamin B12, methylmalonic acid (MMA), holo-transcobalamin (holoTC), and plasma total homocysteine; folate: RBC folate, serum folate; iodine: serum thyroglobulin and urinary iodine. Change in nail zinc concentrations and the composition of the gut microbiome, as well as discretionary salt intake in PSC will be considered secondary outcomes.

## Methods

### Study design

The trial is a community-based, randomized, controlled trial that will enroll and randomize 780 non-pregnant WRA and their households to one of three study groups: 1) FePP-Q5S; 2) eFF-Q5S; or 3) IS. 468 children 12–59 months of age whose mothers are participating in the parent trial will be enrolled into the same three study groups as their mothers. The assigned study salt is intended to meet the needs of the entire household and will be provided to participating women on a monthly basis for a period of 12 months. Biomarkers of MN status will be measured at enrollment, 6 months, and at the end of the 12-month intervention period.

### Study setting

The study will be conducted in approximately 23 semi-rural villages in the district of Mohali in the state of Punjab, India. This site was chosen for the following reasons: 1) The prevalence of anemia among non-pregnant WRA and several MN deficiencies among PSC in the state of Punjab are comparable to or higher than the national average [1, 2]. The recent survey in nearby Ambala district, Haryana also confirmed very high levels of anemia, and iron, folate, and vitamin B12 deficiency [3]. 2) The district of Mohali is easily accessible by the research team, which will make it possible to transport biological samples from the field laboratory to the central laboratory at PGIMER within two hours. 3) Mohali is not currently covered by any state-supported wheat flour fortification or DFS programs.

Villages in Mohali are relatively densely populated and typically consist of 750–2,000 people living in approximately 150–400 households, with approximately one non-pregnant WRA per household. Essentially all households have access to electricity and an improved drinking water source [2]. Furthermore, 89% of women delivered in a health facility and 80% of women had at least four antenatal visits during their last pregnancy. At 98.7%, coverage of IS is excellent. Nonetheless, indicators of maternal and child nutritional status remain suboptimal: 14% of WRA have a body mass index (BMI) < 18.5 kg/m^2^ and 39% are overweight or obese (BMI ≥ 25 kg/m^2^); approximately one-quarter of children under 5 years of age are stunted (height-for-age Z score < -2), and 10% are wasted (weight-for-height Z score < -2).

As part of formative research, a cross-sectional survey was conducted among a representative sample of 100 non-pregnant WRA residing in 11 communities in the study area to confirm the prevalence of MN deficiencies is high, and to measure dietary intake of key MNs and discretionary salt in the baseline diet. A detailed description of this survey is reported elsewhere [17]; however, results indicated that the prevalence of anemia (hemoglobin < 12 g/dL) and iron deficiency (inflammation-adjusted serum ferritin < 15 μg/dL [18]) was 37% and 67%, respectively. The prevalence of zinc deficiency (serum zinc < 70 μg/dL) was 34%, vitamin B12 deficiency (serum vitamin B12 < 150 pmol/L) was 23%, and folate insufficiency (RBC folate < 748 nmol/L) was 70%. The estimated prevalence of inadequate usual dietary intake in the baseline diet, according to the Indian Nutrient Reference Values (NRVs) [19], was 46% for iron, 95% for zinc, 83% for vitamin B12, and 36% for folate. Mean ± SD intake of discretionary salt was 4.6 ± 1.8 g per woman per day.

### Identification and recruitment of participants

**Fig. 1:**
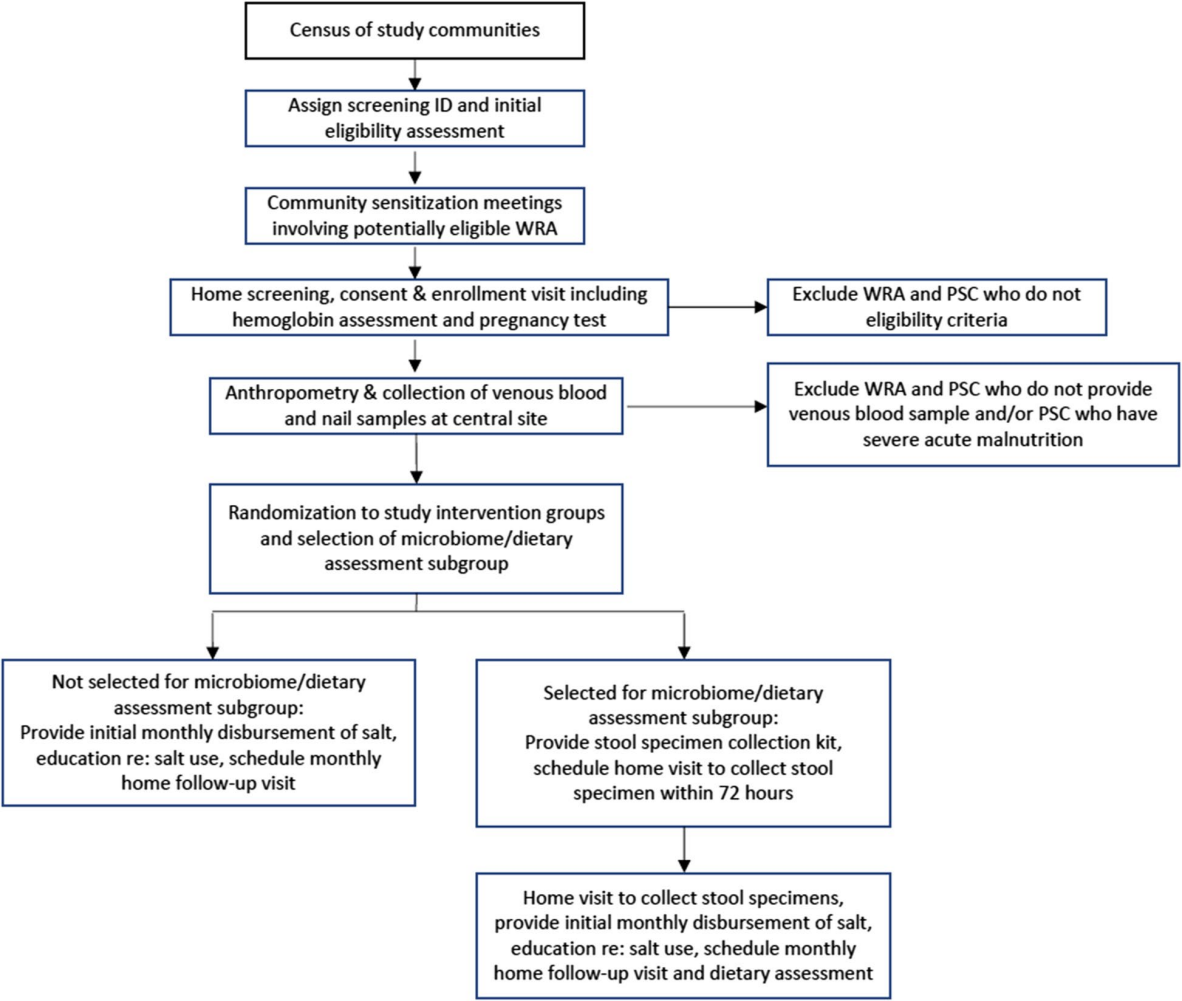
Flow of enrollment and baseline assessments

Figure 1 summarizes the flow of recruitment, enrollment, randomization, and baseline assessment procedures. All relevant government and health officials in the district will be informed about the objective of the trial, eligibility criteria for participation, and study procedures through a series of community sensitization meetings. Research staff will work with village leaders, accredited social health activists (ASHAs), and Anganwadi Workers (AWWs) to inform all households in the study villages about the trial and a census of all study villages will be conducted to establish a sampling frame and identify potentially eligible women and children. Over the next three months, potentially eligible women and their children will be invited to attend weekly informational sessions at a central location in the community where study personnel will describe the public health importance of MN deficiencies in India, the role of fortified foods as a strategy to improve MN intake, the objectives of the Multiply-Fortified Salt (MFS) trial, eligibility criteria for participation, the informed consent procedure, potential risks and benefits of participation, the duration of the study, and the study procedures. Women will have the opportunity to ask questions and will return home to discuss their own participation and their child’s participation with other family members.

Following these meetings women will be visited at home within the next week, at which time a field investigator will formally screen the women and children for eligibility criteria and seek informed consent/parental permission.

### Eligibility criteria

To be eligible for participation in the trial, women must meet the following criteria: 1) 18–49 years of age; 2) Not currently pregnant (confirmed by a urinary human chorionic gonadotropin (HCG) test); 3) Not severely anemic (defined as a hemoglobin concentration < 8.0 g/dL, as measured by HemoCue® 301 +); 4) Permanent resident of the study village with no plans to move or travel outside the village for more than 4 weeks over the next 12 months; 5) No serious health problems that require hospitalization or interfere with eating practices; 6) Willingness to use refined salt provided by the study as a primary source of discretionary salt for the household.

To be eligible for participation in the trial, PSC must fulfill the following criteria: 1) 12–59 months of age; 2) Child’s mother or primary female caregiver has been enrolled into the parent trial; 3) Not severely anemic (defined as a hemoglobin concentration < 7.0 g/dL, as measured by HemoCue® 301 +); 4) Not severely acutely malnourished (defined according to a weight-for-length/height Z-score < -3 or mid-upper arm circumference (MUAC) < 115 mm; 5) No serious medical problems that interfere with the child’s eating practices.

### Enrollment and consent procedures

At the screening and enrollment home visit, the woman and child’s dates of birth will be recorded from their respective birth certificates, Aadhaar identification cards, or health cards to determine exact ages; hemoglobin concentrations will be measured using a HemoCue® 301 + (Ängelholm, Sweden); and the urinary HCG pregnancy test will be administered to women. If the woman or child is found to have severe anemia, they will be referred to the health subcenter for appropriate treatment. If the woman is found to be pregnant, she will be referred to the local ASHA and health center for prenatal care.

Once the field investigator has confirmed all eligibility criteria have been met, consent/parental permission will be obtained. Literate women will be encouraged to read the consent form aloud, under the supervision of the field investigator. Alternatively, because of varying levels of literacy, consent documents will be read to prospective participants in the local language by the field investigator, if necessary. Consent will be acknowledged by signature or thumbprint, and a neutral witness from the community will be present to confirm that the consent procedure was administered appropriately and the prospective participant understood all information.

### Data collection at enrollment

After consent/permission has been obtained and the woman/child has been enrolled in the study, the field investigator will then administer a questionnaire to collect background information on household sociodemographic characteristics; salt procurement, utilization, and practices; food security status (using the Household Food Insecurity Access Scale [20]); and individual dietary diversity, child feeding practices, and recent morbidity. All enrolled women and children will be provided with a container to collect a urine sample and will be scheduled for the biochemistry/anthropometry visit, which will take place at a central location in the village within 3 days of enrollment. Women and children will be asked to appear for this visit having fasted for at least 8 h between the hours of 7am-10am. They will be asked to return their urine samples to the research team at this visit.

### Randomization and blinding

Women and children who meet the eligibility criteria, have provided informed consent, have participated in baseline data collection, and have provided biological specimens and anthropometric measurements (described below) will be individually randomized to receive FePP-Q5S, eFF-Q5S or IS. A randomization list will be prepared by the study biostatistician using block randomization with a block size of 12 to ensure even distribution of the three groups across time. Sealed opaque, numbered envelopes containing a paper with the color-coded group assignment will be prepared. The field investigator responsible for the randomization procedure will spread out the top 12 envelopes and will ask the woman to choose one envelope. After the woman makes her pick, the field investigators will return the envelopes to the pile in numerical order. This process will be repeated until all envelopes in the block have been distributed; then, a new block will be started. For women with participating children, the assigned intervention will de facto apply to both the woman and her child. Each randomized woman and child will receive a color-coded study identification card that matches her assigned intervention group.

After women with participating children have been randomized to one of the three study interventions, a similar block randomization process will be used to randomly select 50 woman-child pairs per group for the dietary assessment/microbiome subsample.

The identity of the color-coded group codes will be stored in sealed envelopes held by an independent researcher not affiliated with the study, and the statistician. The envelopes will be opened and the codes will be revealed only after the statistical analyses of the primary outcomes have been completed and the results have been interpreted.

### Interventions

#### Micronutrient composition of the study interventions

As described above, formative research in the study area indicated that mean discretionary salt intake among non-pregnant WRA in the study area was 4.6 g/day. Although the dietary assessment did not capture dietary intake of PSC, discretionary salt intake of PSC is assumed to be directly proportional to energy intake and the average energy intake of PSC is approximately 50% of the energy intake of WRA. Therefore, the expected discretionary intake of PSC is approximately 2.3 g/day. The SIMPLE macro tool [21] was used to estimate prevalence estimates of inadequate and excessive intakes of each MN in the women’s baseline diet and predict how these estimates would change after the introduction of Q5S at different levels of fortification given the women’s intake of discretionary salt. Because the composition of the MN premix was fixed before the dietary assessment was completed, the level of fortification was adjusted by altering the premix:salt ratio, not the amounts of individual MNs.

Table 1 shows the MN composition of the eFF-Q5S and FePP-Q5S interventions that will be evaluated in the trial. The amount of each MN is shown per gram of salt and as a total daily amount that is expected to be provided by the Q5S assuming average discretionary salt intakes of 4.6 g/day for non-pregnant WRA and 2.3 g/day for children 1–3 years of age. These fortification levels reflect the premix:salt ratio that maximizes the reduction in the prevalence of inadequate intake while ensuring that the prevalence of excessive intake of non-pregnant WRA does not exceed 5% for each MN, in accordance with WHO recommendations [22]. Iodized salt will contain the same amount of iodine as FePP-Q5S and eFF-Q5S, but will not include any other MNs.

**Table 1:** Micronutrient composition of the FePP-Q5S and eFF-Q5S

**Micronutrient**	**Amount of MN per gram of Q5S**	**Total amount of MN provided to NPWRA by Q5S per day**^a^	**Indian NRVs for NPWRA (per day)**			**Total amount of MN provided to children 1-3y by Q5S per day**^b^	**Indian NRVs for children 1–3 y (per day)**		
			**EAR**	**RDA**	**UL**		**EAR**	**RDA**	**UL**
Iron, as ferric pyrophosphate or encapsulated ferrous fumarate	1.3 mg	6.0 mg	15 mg^c^	29 mg	45 mg	3.0 mg	6.0 mg^c^	8.0 mg	40 mg
Zinc, as zinc oxide	1.4 mg	6.4 mg	11 mg^d^	13.2 mg	40 mg	3.2 mg	2.8 mg^d^	3.3 mg^d^	7 mg^e^
Vitamin B12	0.6 μg	2.8 μg	2 μg	2.2 μg	–-	1.4 μg	1.0 μg^f^	1.2 μg^f^	–-
Folic acid	52 μg	239 μg	180 μg^g^	220 μg^g^	1000 μg	120 ug	97 μg^h^	120 μg^h^	^−−−i^
Iodine as potassium iodate	30 μg	138 μg	95 μg	140 μg	1100 μg	69 μg	65 μg	90 μg	300 μg

^a^Assumes an average discretionary salt intake of 4.6 g/day

^b^ Assumes an average discretionary salt intake of 2.3 g/day

^c^The EAR was estimated based on iron bioavailability of 8% accounting for the high phytic acid content of most diets in India

^d^The bioavailability of zinc in Indian diets was estimated to be 23% across all age and sex groups of the Indian population

^e^The Indian NRVs [19] do not specify a UL for zinc for children 1–3 years of age but recommend that the US IOM UL be used. This value is 7 mg/day for children 1–3 years of age

^f^The Indian NRVs [19] specify an EAR and RDA for vitamin B12 among children 6 months-5 years of age. There is no UL for vitamin B12

^g^The EAR and RDA refer to folate. For reference, the IOM and WHO recommended EAR and RDA are 320 μg/d and 400 DFE μg /d, respectively [23]. DFE μg /d is equivalent to food folate + 1.7 × folic acid

^h^The EAR and RDA refer to folate. For reference, the IOM and WHO recommended EAR and RDA are 120 DFE μg/d and 150 DFE μg /d, respectively. DFE μg /d is equivalent to food folate + 1.7 × folic acid

^i^A UL is not specified for children 1–3 years of age. The Indian NRVs [19]specify a UL of 300 μg /day for children 7–9 years of age

#### Production of the interventions

JVS Foods/Wella Nutralogicals in Jaipur, India will be responsible for producing the MN premixes and blending the study salts with technical inputs from researchers at the University of Toronto and ETH Zurich. JVS Foods/Wella Nutralogicals has been approved as a producer of MN premixes by the Food Standards and Safety Authority of India (FSSAI) and adheres to FSSAI standards in its production of IS and DFS. An extrusion process is used to produce the Q5S premixes whereby ferrous fumarate or ferric pyrophosphate plus EDTA, zinc, vitamin B12, and folic acid are extruded in a cold-forming extruder with a small amount of wheat flour (durum semolina) as a binder and vegetable oil as a lubricant through a 400 μm angel hair pasta die. The extruded strands are then placed in a spherulizer to generate spheric balls. Using a sieving screen installed by the output side of the spherulizer, extruded particles in the range of 300–500 μm are collected for further color-masking and coating processes. The coated particles are screened with uniform size of 500–700 μm as the premix to be blended with high-quality, washed, refined salt that has been iodized via spraying with potassium iodate at a ratio of approximately 1 part premix to 60 parts salt in the case of FePP-Q5S and 1 part premix to 77 parts salt in the case of eFF-Q5S. JVS Foods will package the FePP-Q5S, eFF-Q5S, and IS in quantities of 500 g in color-coded, durable polyethylene bags that clearly indicate the salt is for research purposes only. Two colors will be used for each type of salt to prevent possible unmasking. The study salts will be blended and packaged in two production cycles: one approximately two weeks prior to the initiation of enrollment; the second approximately six months after the initiation of data collection.

#### Provision of the interventions to trial participants

At the time of randomization, the woman will be provided with the initial disbursement of her assigned, packaged study salt in a resealable plastic container. Women living in households with 5 members or less will receive 1 kg of salt, those living in households with 6–10 members will receive 1.5 kg of salt, and those living in households with 11 or more members will receive 2 kg of salt. According to our preliminary dietary studies, these quantities should meet the consumption needs of most households for a period of one month. All participating women will be instructed to use their assigned salt to fulfill the cooking and consumption needs of all household members and store the study salt in the plastic containers provided by the research team. Women will also be informed that the study salt may appear slightly different from their usual salt and that these differences should be considered normal and do not reflect a suboptimal product. Although the women will receive information regarding the possible health benefits of Q5S during the sensitization meetings, they will be encouraged to maintain their normal salt consumption practices and not increase their salt intake beyond usual levels. Women will also receive explicit instructions not to share their assigned salt with any people outside their household; procure any salt from outside sources; sell, trade or share any extra salt that is not consumed by members of their household. Women will be instructed to contact a member of the research team for a supplemental disbursement of their assigned study salt, in the rare event that they deplete their allotment prior to the next scheduled monthly follow-up visit.

### Visit schedule

Table 2 summarizes the timing of various data collection procedures at enrollment and throughout the 12-month follow-up period. Briefly, field investigators will conduct the screening and enrollment visit in the woman’s home; however, women and children will be requested to visit a central location in the village for the collection of biological samples, performance of anthropometric measurements, and randomization to a study intervention within 3 days of enrollment. Collection of biological specimens and performance of anthropometric measurements will be repeated at 6 months and upon completion of the 12-month intervention period. As noted above, field investigators will visit the women in their homes on a monthly basis throughout the intervention period to collect unused salt, assess adherence, monitor morbidity, and provide the next disbursement of the assigned study salt. Independent members of the research team who are blinded to the group assignments will also conduct structured interviews with a random subgroup of 25 participants in each intervention group on a quarterly basis to obtain qualitative data on the acceptability of the study salts. Over the course of the data collection period, dietary field investigators will also conduct a detailed dietary assessment to assess intake of discretionary salt in a randomly selected subgroup of 50 PSC participants from each intervention group, as described below.

**Table 2:** Schedule of data collection activities over the intervention period

		**Month of follow-up**											
**Activity**	**Enrollment**	**1**	**2**	**3**	**4**	**5**	**6**	**7**	**8**	**9**	**10**	**11**	**12**
Background sociodemographics; food security status, two-week child morbidity recall	x												
Amount of salt used and amount of salt distributed		x	x	x	x	x	x	x	x	x	x	x	x
Acceptability of study salt, morbidity		x	x	x	x	x	x	x	x	x	x	x	x
Structured interviews to obtain detailed acceptability data^a^				x			x			x			x
Child dietary assessment^b^		x	x	x	x	x	x	x	x	x	x	x	x
Laboratory analyses of salt samples				x			x			x			x
Anthropometric measurements	x						x						x
Capillary blood draw for hemoglobin measurement	x						x						X
Venous blood draw for assessment of malaria, CBC, serum zinc, ferritin, sTfR, CRP, AGP, vitamin B12, folate, holoTC, MMA, thyroglobulin, plasma homocysteine, RBC folate	x						x						x
Urinary iodine and creatinine concentrations	x						x						x
Stool samples^b^	x												x
Fingernail samples	x												x

^a^Subgroup of 25 women per group

^b^Subgroup of 50 mother–child pairs per group

### Biological specimen collection, processing and analysis

#### Collection of biological specimens

At the biochemistry/anthropometry visit, trained, local phlebotomists with pediatric experience will draw a total of 10 mL of venous blood from each participating woman and 7 mL from each participating child into one zinc-free red serum monovette, and one violet plasma monovette with K3 EDTA using 21/23 gauge multiflies (Sarstedt, Numbrecht Germany), recording the time of blood collection and time of last meal, and following International Zinc Nutrition Consultative Group (IZiNCG) recommendations for the collection and processing of samples for serum zinc [24]. The blood samples will then be placed in a portable, electronic, temperature-controlled cooler for later transport to the field laboratory. The phlebotomist will also measure hemoglobin concentrations from a finger-prick capillary blood sample using a HemoCue**®** 301 + instrument.

As part of the biochemistry/anthropometry visit, a repeat urine sample will also be collected from as many participants as possible to enable estimation of the prevalence of inadequate iodine intake [25, 26]. Women and older children will be asked to urinate into a specimen collection container, whereas a urine collection bag will be placed on younger children, if needed. Urine samples will be transferred into screw-top polyethylene vials by a field investigator for transport to the field laboratory and later analysis of urinary iodine and creatinine concentrations.

At least two fingernail and toenail clippings approximately 5 mm in length will be cut from each participant using stainless steel safety nail clippers following IZiNCG recommendations and stored in Ziploc bags at room temperature in an airtight container [27].

Women and children assigned to the dietary assessment/microbiome subgroup will be provided with a stool sample collection and preservation kit and will be scheduled for a home visit within the next 3 days, during which they will provide the collected stool sample. Women and children in this subgroup will receive the initial disbursement of their assigned study salt at this home visit after they have provided the stool sample.

#### Processing of biological specimens

Upon arrival at the field laboratory, violet plasma monovettes will immediately be inserted into a hematology analyzer for CBC measurement. Then 100 μL of whole blood will be aliquoted into a cryovial containing 1% ascorbic acid for later analysis of RBC folate. The remaining whole blood in the violet plasma monovette will be centrifuged at 2000 × g for 10 min to separate plasma from red blood cells, and the plasma will be aliquoted to cryovials for analysis of homocysteine. Whole blood samples in the red serum monovettes will be left undisturbed for 30 min to allow the blood to clot, after which time they will be centrifuged at 200 × g for 10 min. Serum will then be aliquoted into cryovials for later analysis of zinc, ferritin, soluble transferrin receptor (sTfR), AGP, CRP, folate, vitamin B12, MMA, holoTC and thyroglobulin. Trace element-free supplies will be used and IZiNCG recommendations will be followed avoid contamination [24]. All aliquoted samples will be stored temporarily in the field laboratory at -20 °C and later transported to the PGIMER central laboratory for storage at -80 °C prior to analysis or shipment.

#### Laboratory analysis of biological specimens

Primary analysis of serum zinc concentrations will be performed in a laboratory with certified procedures and accurate and reproducible results for zinc analyses using Inductively Coupled Plasma-Mass Spectrometry (Agilent 7500X, ICP-MS). Seronorm Trace Elements Serum Level 1, catalog #201,405 will be used as an internal control.

Serum holoTC and MMA will be analyzed at St. John’s Research Institute in Bangalore, India. Serum holoTC will be determined with the Axis-Shield HoloTC ELISA (Axis Shield Diagnostics Ltd.). Serum MMA will be analyzed with the use of ultraperformance liquid-chromatography-tandem mass spectrometry [28].

Analysis of RBC folate; plasma homocysteine; serum ferritin, sTfR, CRP, AGP, folate and vitamin B12; urinary iodine and creatinine concentrations; and the gut microbiome will be performed at PGIMER. Analysis of RBC folate and serum folate will be carried out using the microbiologic assay with chloramphenicol-resistant strains of *Lactobacillus rhamnosus* culture and a 5-methyltetrahydrofolate calibrator [29]. Quality control pools for low, medium and high whole blood pools will be performed and aliquots will be stored at – 80 °C for use in each assay. For each run, a standard growth curve of the organisms will be carried out. Four replicates will be run for each individual sample to assure generation of accurate results. The growth of the organisms will be read as turbidity after incubating at 37 °C for 40–42 h. Serum vitamin B12, thyroglobulin, CRP, and ferritin will be assessed on Cobas e411 by chemiluminescence (Roche Diagnostics, Germany). Serum CRP, sTfR and AGP concentrations will be measured on Cobas c systems using a particle enhanced immunoturbidimetric assay (Roche Diagnostics, Germany). Plasma total homocysteine will be determined by the fully automated ADVIA Centaur XP (Siemens).

Urinary creatinine concentrations will be assessed by kinetic colorimetric assay based on the Jaffé method on an automated Roche Cobas system. Urinary iodine concentrations will be measured using an enzyme-linked immunosorbent assay (ELISA).

Fecal samples will be analyzed for the enteric microbiome using 16S rRNA sequencing. Gene-specific primers will be used to amplify the 16S V3 and V4 regions. The libraries will be assessed for quality using Bioanalyzer DNA kit (Agilent Technologies, Inc., USA), and will be quantified using fluorometric quantification for dsDNA binding dyes. The normalized libraries will be pooled and sequenced on MiSeq Sequencing System (Illumina Inc, USA).

Fingernail samples will be manually scraped with a ceramic blade to remove debris and placed into microcentrifuge tubes to reach a mass of 3–10 mg. Samples will then be cleaned to remove external zinc contamination using a modified version of the cleaning protocol recommended by IZiNCG [27]. After cleaning, the samples will be dried, weighed and then digested in OmniTrace 70% HNO3 for 12–18 h. Samples will then be diluted to a final concentration of 5% HNO3 and centrifuged at 3000 × g for 10 min prior to analysis using Inductively Coupled Plasma-Optical Emission Spectroscopy (ICP-OES) at the University of California San Francisco as previously described [30]. Nail zinc concentrations will also be analyzed using portable X-ray fluorescence at Mount Allison University in Canada as previously described [31].

#### Anthropometric measurements

Anthropometric measurements will be performed by a team of two trained anthropometrists following standard procedures at the biochemistry/anthropometry visit shortly after enrollment, at 6 months, and 12 months [32]. All women and children 24–59 months of age will have their height measured to the nearest 0.1 cm using a stadiometer (SECA). Children who are 12–23 months of age at enrollment will have their length measured to the nearest 0.1 cm using an infantometer (SECA) at all three visits. A digital balance (SECA) will be used to measure the weight of all women and children to the nearest 10 g. The MUAC of all women and children will also be measured to the nearest 1 mm using flexible, non-stretchable measuring tapes. Anthropometrists will use WHO field tables to determine the child’s weight-for-length (WLZ) or weight-for-height Z-score (WHZ). If a child is identified as having severe acute malnutrition, defined as a WLZ or WHZ < -3.0, a MUAC < 115 mm, and/or bipedal edema, he/she will be referred to the health subcenter for appropriate treatment and excluded from the trial.

#### Monthly follow-up visits

Field investigators will contact participants by phone one week after randomization to ensure instructions regarding the study salt are understood and to resolve any concerns that may have arisen. With support from village ASHAs, the field investigators will visit the household of each participating woman/child on a monthly basis to collect and record any unused salt that was previously provided; collect general information on acceptability, usage, and consumption; assess morbidity in the preceding week; provide the next monthly disbursement of salt; and promote continued participation in the trial. These monthly distribution visits will occur throughout the 12-month follow-up period.

### Dietary assessment of preschool children

#### Collection of dietary data

As depicted in Table 2, a dietary assessment of 50 randomly selected children per study group will be conducted over the course of the study implementation period. The objective of these assessments will be to measure the child’s discretionary salt intake and corresponding exposure to the study intervention. Trained dietary field investigators will simultaneously collect a one-day duplicate diet composite and a one-day weighed food record from each of the 150 children in their homes. Selection of the days for the diet assessment will ensure that weekend days, market days, and weekdays are proportionately represented to account for any day-of-the-week effects on food and/or nutrient intakes in the group. A second diet record and duplicate diet will be collected from a random subgroup of 20 children in each group on a non-consecutive day to assess for intra-individual variability in intake.

Prior to the scheduled day of the diet assessment, each child’s mother will be provided with a small stipend to cover the cost of extra portions of food that are equivalent to what the child would typically consume. The mother will be instructed to prepare this increased quantity of food on the day of the diet assessment. On the assigned assessment day, upon arrival at the child’s home, the dietary field investigator will take a sample of the refined salt that the mother will use to cook and flavor all foods and beverages over the course of the day for later analysis of iodine content. Throughout the day, a duplicate sample of each food and beverage “as eaten”, including an equivalent amount of salt that may have been added to the child’s portion at the table, and the same weight as that consumed by the child will be weighed using dietary scales (precise to the nearest 0.1 g), recorded, and transferred to a polyethylene container.

The amount of salt added to all foods (including recipes for mixed dishes consumed by all members of the households) and beverages before, during, and after cooking will also be measured to the nearest 0.1 g using the dietary scale. For mixed dishes that contain salt and are consumed by multiple members of the household, the proportion of the total dish consumed by the child will be measured. After collection, the diet composites and salt samples will be transported in the cooler box to the field laboratory for processing within 24 h of collection.

The analysis of duplicate diet composites will be used to estimate the average habitual intake of the study salt in two ways. The first method will involve the quantification of the iodine content of the duplicate diet composite. Since the study salt will likely provide a major source of iodine in the child’s diet, and the iodine content of each child’s salt sample will be analyzed, it will then be possible to estimate the amount of study salt consumed on the day of the diet assessment. The weighed food records will be reviewed to identify any other potential dietary sources of iodine. Samples of these other foods and beverages will be collected as necessary, analyzed for iodine, and appropriately subtracted from the total iodine content of the duplicate diet composite. The second method of estimating average study salt intake will be to quantify the sodium content of the duplicate diet composite. Since this value will represent the total sodium intake of the child (i.e. the sodium content of all foods consumed plus sodium from the study salt), it will be necessary to estimate the amount and/or proportion of sodium contributed by the study salt vs. other dietary sources. To perform this estimation, a research assistant will prepare “replicate” diet composites using data from the weighed food records of 60 children without adding any study salt. By subtracting the amount of sodium in the “replicate” diet composites that are free from study salt from the total sodium content of the original diet composite, it will be possible to estimate the typical average amount and corresponding proportion of total sodium that is contributed by the study salt.

#### Processing and laboratory analysis of the duplicate diet composites

At the field laboratory, the weighed diet composites will be blended to a homogenous slurry using a large-capacity glass blender, after which 50 mL aliquots will be withdrawn into trace element-free polyethylene containers. These samples will be stored at -20 °C and then transported to the National Institute of Food Technology, Entrepreneurship and Management (NIFTEM) in Haryana, India. The concentration of iodine in the samples will be analyzed in duplicate using ICP-MS by hot block extraction and tetramethyl-ammonium hydroxide digestion (Sigma Aldrich). Samples for sodium analysis will be prepared using acid digestion and analyzed in duplicate using Inductively Coupled Plasma-Optical Emission Spectrometry (ICP-OES).

### Data management and analysis

#### Data collection and management

Data will be collected electronically using REDCap, a secure, cloud-based mobile platform. Data collection forms were developed by research staff and include pre-determined logic checks and range validations. Forms will be deployed onto Samsung tablets which are equipped for mobile internet access. After the field investigator has completed a data collection form, a temporary version will be synchronized with a central Amazon Web Server. At the end of each day of field work, the supervisor will be responsible for verifying each temporary form for completeness and accuracy and uploading a final version to the server. The supervisor will also conduct repeat enrollment and/or follow-up visits for one randomly selected participant per field investigator each week. The data manager will implement a data monitoring protocol on a weekly basis to track enrollment progress, identify delays in data submission, flag errors related to study identification numbers, identify possible concerns regarding adherence to the study intervention. Deidentified temporary datasets will be securely transferred to the University of California San Francisco (UCSF) server on a weekly basis; final datasets will be transferred at the end of the data collection period.

#### Sample size

The sample size calculation was based on the objective of detecting an effect size of 0.3 standard deviation (SD) units for the change in each of the primary biomarkers among non-pregnant WRA participants from baseline to the end of the 12-month intervention period. Assuming an alpha of 0.05, power of 80%, three-group study design, and attrition rate of 10%, 240 non-pregnant WRA will be required per group. Based on MN status data from the formative survey conducted in Mohali district, an effect size of 0.3 SD units translates into between group differences of approximately 0.45 g/dL for hemoglobin, 4.7 mg/L for ferritin, 3.3 µg/dL for serum zinc, 48.4 pmol/L for serum vitamin B12, and 123 nmol/L for RBC folate. These effect sizes are comparable to or slightly smaller than those observed in Cameroonian women 12 months after the introduction of fortified wheat flour [33]. Given the crude birth rate of 14.1 per 1,000 people per year in Punjab, approximately 20 births are expected to occur among WRA in each study group over the intervention period. Therefore, 260 WRA who are confirmed as non-pregnant will be enrolled with the expectation that only 240 of these WRA will remain non-pregnant throughout the 12-month intervention period.

Based on demographic data from Mohali, approximately one-half of all non-pregnant WRA in the study are expected to have at least one child in the eligible age range (i.e. 12–59 months of age). Based on results of the CNNS, only 1.2% of these children are expected to be ineligible due to severe anemia [1]. Recognizing the likelihood that the attrition rate may be higher among PSC than WRA, we will enroll 156 PSC into each arm of the trial with a goal of obtaining an effective sample size greater to or equal than 130 PSC per arm for pairwise group comparisons. This sample size will enable the detection of a change in the prevalence of anemia from 40 to 23% of iron deficiency from 67 to 49%, of zinc deficiency from 21 to 8%, of vitamin B12 deficiency from 17 to 5% and of folate deficiency from 10 to 1%, assuming an alpha of 0.05, power of 80%, and attrition rate of 20% [1]. These calculations translate into an effect size of 0.35 SD units for the change in the mean concentration of the primary MN biomarkers from enrollment to the end of the 12-month intervention period.

#### Outcomes

Primary outcomes of the trial are the change in the mean concentration of the following MN biomarkers, and the corresponding change in the prevalence of each MN deficiency among WRA and PSC from enrollment to 12 months: iron: serum ferritin and sTfR; anemia: hemoglobin; zinc: serum zinc; vitamin B12: serum vitamin B12, MMA, holoTC, and plasma total homocysteine; folate: serum folate, red blood cell folate; iodine: serum thyroglobulin, and group-specific median urinary iodine concentration.

Secondary outcomes include change in mean nail zinc concentrations among a subsample of WRA and PSC from enrollment to 12 months; change in the composition, richness, and diversity of the major phyla and genera of the gut microbiome among a subsample of WRA and PSC from enrollment to 12 months; discretionary intake of salt among PSC.

#### Definition of outcome variables

MN deficiencies among non-pregnant WRA will be defined according to the following cut-offs: 1) anemia: hemoglobin < 12 g/dL; 2) iron deficiency: inflammation-adjusted ferritin < 15 μg/dL [18] or sTfR ≥ 8.3 mg/L [34]; 3) iron deficiency anemia: hemoglobin < 12 g/dL and either inflammation-adjusted ferritin < 15 μg/dL or sTfR ≥ 8.3 mg/L; 4) hypozincemia: fasting serum zinc < 70 μg/dL; 5) vitamin B12 deficiency: serum vitamin B12 < 150 pmol/L; 6) vitamin B12 insufficiency: serum vitamin B12 150–221 pmol/L; 7) folate deficiency: RBC folate < 317 nmol/L; 8) folate insufficiency: RBC folate < 748 nmol/L. Cut-offs of < 40 pmol/L and > 0.376 μmol/L will be used to define low holoTC and elevated MMA, respectively. A combined B12 indicator will also be calculated and compared across groups as previously described [7, 35].

Among PSC, MN deficiencies will be defined according to the following cutoffs: 1) anemia: hemoglobin < 11 g/dL; 2) iron deficiency: inflammation-adjusted ferritin < 15 μg/dL or sTfR ≥ 8.3 mg/L; 3) iron deficiency anemia: hemoglobin < 11 g/dL and either inflammation-adjusted ferritin < 15 μg/dL or sTfR ≥ 8.3 mg/L; 4) hypozincemia: fasting inflammation-adjusted serum zinc < 65 μg/dL [36]; 5) vitamin B12 deficiency: serum vitamin B12 < 201 pmo/L; 6) vitamin B12 insufficiency: serum vitamin B12 150–221 pmol/L; 7) folate deficiency: serum folate < 10 nmol/L, RBC folate < 340 nmol/L. Holotranscobalamin concentrations of < 12 pmol/L and < 19 pmol/L will be used in children aged 12–24 months and 24–59 months, respectively; methylmalonic acid concentrations > 300 nmol/L will be used in children 12–59 months of age; and homocysteine concentrations > 13 μmol/L will be used to define low vitamin B12 status. A median urinary iodine concentration < 100 μg/L will be defined as inadequate [37]. Equations to convert urinary sodium concentrations and urinary creatinine concentrations to predicted-urinary sodium excretion will be applied as described by Rios-Leyvraz et al. [38].

#### Data analysis principles

A participant flow diagram will be prepared in accordance with the 2010 CONSORT guidelines. For each set of analyses, statistical analysis plans (SAPs) will be prepared and publicly posted before analysis is conducted. Primary data analyses will be conducted in accordance with the plan described below using SAS version 9.4 (SAS institute, Cary, NC, USA). The primary analysis will be conducted on a “complete-case intention to treat” basis whereby results for all participants will be analyzed according to the study group to which they were randomized. Data from participants who were lost to follow-up will be included in the analysis if available. If a substantial portion of participants are lost to follow-up (> 20%) then an additional sensitivity analysis will be conducted using a missing data approach such, as multiple imputation or inverse probability of censoring-weighted analysis, as pre-specified in the relevant SAP. A secondary set of analyses will also be conducted among the sub-set of participants who adhered well to the protocol (i.e. a “per protocol” analysis). All statistical analyses will be two-sided with a *p* value < 0.05 indicating statistical significance unless otherwise pre-specified in statistical analysis plans. In cases where more than 10% of observations are missing for a dependent variable, we will report the number of observations used in the analysis. We will also compare the baseline characteristics of participants who are lost to follow-up with those who remained in the trial for the duration of the follow-up period to identify any possible biases and include such characteristics as covariates in the statistical analysis if bias is detected. To limit potential bias and ensure blinding throughout the statistical analyses and interpretation of results, biological specimens and all participant data will contain only the participant’s study identification number and a numerical code (not the color code) to represent the group assignment. All intervention groups will remain masked until all statistical analyses of the primary outcomes have been completed. Formal interim analyses that examine study progress for futility issues will not be conducted.

#### Data analysis plan

Box plots and/or histograms of individual continuous variables will be generated to visually check for outliers. Scatterplots of related variables will also be generated. Outliers that are clearly impossible or implausible values will be corrected if possible or recoded to missing if correction is not possible. Outliers that are plausible or possible will be maintained. All variables measured at the time of enrollment will be considered background or baseline characteristics. These baseline characteristics will be presented in a table, by intervention group.

The change in the concentration of each biomarker between enrollment and 6 months, and enrollment and 12 months will be compared between groups using analysis of covariance (ANCOVA, SAS GLM procedure) with baseline MN biomarkers as covariates in the models. Linear mixed models (SAS MIXED procedure) will also be used to compare repeated measures of these continuous variables over the three time points. These models will employ fully conditional specification methods to impute values for which biochemical outcome data are missing. These models will include any baseline characteristics that were not equally distributed between study groups at enrollment or which can be associated with the outcome. They will also include a variable that reflects the village in which the participant resided.

To estimate the average intake of study salt from the iodine content of the duplicate diet composite, the amount of iodine from any known dietary sources of iodine (other than the study salt) will be subtracted from the total iodine content of the duplicate diet composite. The difference will then be divided by the measured concentration of iodine in the study salt sample that was obtained from the child’s household. To estimate the intake of study salt from the sodium content of the duplicate diet composite, the sodium content of each of the “example” duplicate diets will be subtracted from the total sodium content of the original duplicate diet in the 60 children that have both sets of data available. The difference will then be multiplied by 2.5419, which reflects the molar mass of sodium in salt (i.e. sodium chloride). The average proportion of sodium contributed by dietary sources other than the discretionary salt can be applied to the other 60 children to obtain a more precise estimate of habitual intake of discretionary salt. The University of California, Davis/NCI SIMPLE macro [21] will be used to account for intra-subject variability in intakes estimated from the 60 children (20 per group) who provided duplicate diet composites on two days, and to adjust the observed distribution of salt intake to the corresponding usual intake distribution.

### Safety and ethics

#### Data safety monitoring

Adverse events will be monitored through the monthly follow-up visits and through ad hoc phone reporting to research staff. All suspected adverse events will be documented and reviewed by a study physician, and reported according to standard operating procedures of the UCSF Institutional Review Board (IRB) and PGIMER Institutional Ethical Committee (IEC). Serious adverse events (SAEs, e.g. deaths, hospitalizations (i.e. an overnight stay in the hospital because of illness), and severe acute reactions associated with consumption of the study salt) will be reported at regular intervals to the UCSF and PGIMER IRBs.

#### Ethical and institutional approval

Ethical approval of the trial has been obtained from the UCSF IRB and the PGIMER IEC. The trial has been registered with clinicaltrials.gov (NCT05166980) and the Clinical Trials Registry-India (CTRI/2022/02/040333 and CTRI/2022/02/04032). The trial protocol also received approval from India’s Health Ministry Screening Committee (2022–16,415/F1 and 2021–16,172/F1). Amendments to the study protocol will be submitted to the UCSF IRB and the PGIMER IEC, and the trial registries will be updated accordingly.

#### Confidentiality

Privacy, anonymity and confidentiality of all data/information identifying the participants will be strictly maintained. All medical information and results of the laboratory tests performed on the participants will be kept confidential. Only de-identified data will be securely transmitted to UCSF servers, as described above. Participants and/or parents or legal guardians of PSC participants will be able to freely communicate with the local Principal Investigators of the study (contact information will be provided on the consent form).

### Trial organization

The MFS trial is led by Dr. Christine McDonald (UCSF) and Drs. Reena Das and Mona Duggal (PGIMER). Technical support and input into various aspects of the study design, study protocol, laboratory analyses, and statistical analyses has been provided by members of the International Zinc Nutrition Consultative Group (IZiNCG) Steering Committee, and several co-investigators/collaborators at the University of California, Davis, University of Colorado, University of Otago, University of Toronto, ETH Zurich, Subharti Medical College, St. John’s Research Institute, and the National Institute of Food Technology Entrepreneurship and Management (NIFTEM). One UCSF postdoctoral research fellow has been hired as scientific field coordinator to manage and supervise implementation of the trial activities.

A large team of field- and laboratory-based staff has been hired by PGIMER and will implement the various activities according to the study protocol. This team consists of one project coordinator, one research coordinator, one accountant, one junior administrative officer one field supervisor, six field investigators, three dietary field investigators, one lab supervisor, two senior and three junior phlebotomists/laboratory technicians, one data scientist, one data manager, two drivers/field attendants, two security guards, and one cleaner.

Intensive training of all research staff will be conducted prior to the initiation of field activities. This will include didactic review of the study protocol and standard operating procedures (SOPs), pilot testing of all research activities in the field, revision of the SOPs as necessary, and refresher training.

The PGIMER co-PIs will make weekly visits to the field throughout the data collection period to supervise research activities and meet with field staff and local stakeholders. All senior members of the research team based in the US and India will hold virtual team meetings on a weekly basis to track progress and discuss important issues pertaining to the implementation of the trial.

## Discussion

Salt fortification has been implemented as a public health intervention in India for nearly 40 years. In 1983, it was decided that all edible salt in India would be iodized by 1992 as a strategy to prevent iodine deficiency disorders [39]. At that time, the prevalence of neonatal hypothyroidism had reached 13.3% in iodine-deficient regions and the impact of iodine deficiency on early brain development, cognition and learning abilities of children were becoming more apparent [40]. Since then, the incidence of iodine deficiency disorders has declined dramatically, coverage of IS has reached 94% [2], and India has been classified as having adequate iodine intake. Salt has been recognized as an appropriate fortification vehicle for iron in addition to iodine, and standards have been developed for the production of DFS by FSSAI. DFS now reaches more than 75 million people through state-supported programs and millions more through private sector markets [41]. Several studies have demonstrated the efficacy of DFS in improving iron status while preserving the beneficial effects on iodine status. A recent meta-analysis that included 12 efficacy trials (8 of which were conducted in India) showed that DFS reduced the risks of anemia and iron deficiency by 41% and 63%, respectively [14]. Stratified analyses revealed that these positive effects were seen among WRA and school-aged children.

Although advances in extrusion and encapsulation technology have enabled additional MNs to be included as fortificants [15], limited research has evaluated the nutritional impact of MFS among nutritionally vulnerable populations, and concerns remain regarding the optimal form of iron. This trial will narrow these critical gaps in the current evidence base. It will also be the first trial to examine the effects of MFS on the MN status of children 12–59 months of age, a group that has been excluded from many of the previous trials of DFS. If proven efficacious, MFS has the potential to drastically reduce the burden of MN deficiencies in India, and around the world. Although effectiveness will be needed to examine the impact of MFS under real world conditions, salt fortification will piggy-back on existing platforms to produce IS DFS, making it possible to scale-up the intervention quickly.

## Trial status

Enrollment into the trial had not commenced at the time of initial submission of the manuscript. Enrollment is anticipated to commence in the summer of 2022.

## Data Availability

The complete dataset and study forms will be made available online at osf.io 3 years after the completion of data collection.

## Abbreviations

MN: Micronutrients
WRA: Women of reproductive age
PSC: Preschool-aged children
eFF-Q5S: Quintuply-fortified salt with iron in the form of encapsulated ferrous fumarate, zinc, vitamin B12, folic acid, and iodine
FePP-Q5S: Quintuply-fortified salt with iron in the form of ferric pyrophosphate plus EDTA, zinc, vitamin B12, folic acid, and iodine
CNNS: Comprehensive National Nutrition Survey
RBC: Red blood cell
NFHS: National Family Health Survey
NTD: Neural tube defects
DFS: Doubly-fortified salt, i.e. salt fortified with iron and iodine
PDS: Public Distribution System
ICDS: Integrated Child Development Services
EDTA: Ethylenediaminetetraacetic acid
IS: Iodized salt
MMA: Methylmalonic acid
holoTC: Holo-transcobalamin
BMI: Body mass index
ASHA: Accredited social health activists
AWW: Anganwadi Workers
HCG: Human chorionic gonadotropin
MUAC: Mid-upper arm circumference
NRV: Nutrient reference value
EAR: Estimated average requirement
RDA: Recommended dietary allowance
UL: Tolerable upper intake level
CRP: C-reactive protein
AGP: Alpha-1-acid glycoprotein
UCSF: University of California San Francisco
PGIMER: Postgraduate Institute of Medical Education and Research, Chandigarh
